# Examining shortened versions of the Social Responsiveness Scale for use in autism spectrum disorder prediction and as a quantitative trait measure: Results from a validation study of 3–5 year old children

**DOI:** 10.1002/jcv2.12106

**Published:** 2022-10-05

**Authors:** Kristen Lyall, Juliette Rando, Bridget Toroni, Tobechukwu Ezeh, John N. Constantino, Lisa A. Croen, Brigid Garvin, Kate Piselli, James Connell, Aaron J. Kaat, Craig J. Newschaffer, program collaborators for Environmental influences on Child Health Outcomes

**Affiliations:** ^1^ AJ Drexel Autism Institute Drexel University Philadelphia Pennsylvania USA; ^2^ Department of Epidemiology and Biostatistics Dornsife School of Public Health Drexel University Philadelphia Pennsylvania USA; ^3^ Department of Psychiatry Washington University St. Louis Missouri USA; ^4^ Division of Research Kaiser Permanente Oakland California USA; ^5^ School of Education Drexel University Philadelphia Pennsylvania USA; ^6^ Department of Medical Social Sciences Feinberg School of Medicine Northwestern University Chicago Illinois USA; ^7^ College of Health and Human Development Pennsylvania State University Philadelphia PennsylvaniaPA USA

**Keywords:** autism spectrum disorder, quantitative traits, Social Responsiveness Scale, validation study

## Abstract

**Background:**

The Social Responsiveness Scale (SRS) is a 65‐item measure yielding a continuous score capturing autism‐related traits. Scores based on SRS item subsets have been analytically examined but administration of shortened versions has not been evaluated prospectively.

**Objective:**

The goal of this study was to compare psychometric properties of two shortened versions of the SRS to the full 65‐item SRS, in young children from both a clinical and general population setting.

**Methods:**

Study participants (aged 3–5 years) were drawn from the AJ Drexel Autism Institute clinic (*n* = 154) and Kaiser Permanente Northern California (*n* = 201) and block randomized to receive either the 16‐item short SRS, a newly developed computer adaptive testing‐SRS, or the published full‐length SRS. Total scores across the three SRS administration methods were scaled to facilitate comparisons. Scores were plotted to assess distributional properties, while Receiver Operating Characteristic analysis was used to estimate Area Under the Curve (AUC) and address predictive ability.

**Results:**

Overall, distributional properties of the three administration methods were highly comparable, with shortened measures demonstrating similar ability to capture the range of the distribution and case non‐case separation as the full SRS. In addition, AUC values were high (0.91–0.97) and comparable across the administration methods, though there was evidence of difference in predictive ability across measures for females (AUC for full SRS = 0.99 vs. 0.84 for short). Within individual comparisons of short versus full scores (available only for participants at the general population site) suggested underestimation of actual full SRS scores with the CAT‐SRS.

**Conclusions:**

Our findings broadly support the construct validity and performance of shortened SRS versions examined here, though the full measure may be needed to more accurately assess traits consistent with ASD diagnosis in females. This work suggests opportunities for collection of ASD‐related phenotype in settings where participant burden or feasibility considerations may have otherwise prohibited such measurement.

**Key points**

- Quantitative measurement of social communication traits is important for characterizing functioning across the population.
- Prior work proposed a shortened version of the Social Responsiveness Scale (SRS). Properties of this shortened version have not been examined in a field validation study, nor has prior work considered computer adaptive testing (CAT) administrations.
- We conducted a validation study across a clinical and general population site, including approximately 200 participants each, randomized to one of the 3 versions (short 16‐item SRS, full published 65‐item SRS, or CAT‐SRS).
- Predictive ability and distributional properties were highly comparable across the 3 versions, supporting comparability of shortened and full SRS scores.
- Findings suggest opportunities to increase efficiency and broaden collection on quantitative social communication traits.

## INTRODUCTION

Autism spectrum disorder (ASD) is a neurodevelopmental condition that spans a range of functioning. Evidence suggests that core features of ASD, including social communication traits, extend into the general population and can be measured along a continuum (Constantino & Todd, 2003, 2005; Lyall et al., 2014; Robinson et al., 2011a). One of the most widely used measures of this continuum of broader autism phenotype is the Social Responsiveness Scale (SRS) (Constantino & Gruber, 2012). The measure has been previously validated against gold standards for ASD diagnosis, and demonstrated strong psychometric properties (Bölte et al., 2011; Charman et al., 2007; Constantino et al., 2003; Constantino & Gruber, 2012). There is also evidence that SRS scores and other quantitatively‐assessed ASD traits demonstrate comparable associations with known risk factors for ASD (including examples across neurobiologic, familial and genetic, and environmental factors) as those observed for the diagnosis (Constantino & Todd, 2005; Lundstrom et al., 2012; Lyall et al., 2022; Risch et al., 2014; Robinson et al., 2011b), providing further support for construct validity. When considering use as a quantitative trait measure, the SRS also has strong distributional properties, demonstrating utility and functionality in capturing the broader phenotype across the general population, and these features also align with recent calls to incorporate Research Domain Criteria approaches (Constantino & Todd, 2003; Cuthbert & Insel, 2013; Lyall et al., 2014).

While the SRS is not considered a diagnostic tool, nor was it intended to be, it does enable screening and assessment of ASD‐related phenotype more efficiently, and therefore often more feasibly and affordably for large‐scale research, than clinical assessments. Nonetheless, for research studies with extensive batteries seeking to assess child health and development across multiple domains, and often also collecting data on host of environmental, social, and contextual factors that may influence these outcomes, there is a practical need for efficiency across assessments. This consideration, combined with some questions as to the influence of other factors on SRS scores, led to work seeking to develop an abbreviated version of the SRS. Specifically, Sturm and colleagues developed a 16‐item version of the SRS based on item response theory (IRT) analyses using existing 65‐item SRS data from several autism databases (Simons Simplex, Interactive Autism Network, the National Database for Autism Research, and Autism Genetic Resource Exchange) (Sturm et al., 2017). From these analyses, 16 items were selected to optimize social communication assessment based on high factor loadings and low evidence for differential functioning by age, sex, and expressive language, as well as expert consideration of content validity. However, no prior study to date has prospectively examined the measure as a stand‐alone questionnaire in a field validation study, and limited work has considered whether the shortened version's development in an autism‐skewed sample impacts its performance as a quantitative trait measure in the general population.

We sought to address these questions in the present validation study. Our goals were to examine the distributional properties of the 16‐item shortened version of the SRS, relative to the full 65‐item version, in order to address performance as a quantitative trait measure in capturing variability across the population, as well as to assess the shortened version's predictive ability, in both a clinical sample as well as a sample drawn from the general population. In addition, in order to address whether there are additional ways to shorten and optimize the efficiency of SRS administration, we also examined performance of a newly developed computer adaptive testing (CAT) version of the SRS (Kaat et al., manuscript).

## METHODS

### Study population

Recruitment took place at a clinical site at Drexel University in Philadelphia, Pennsylvania, and a general population site consisting of two San Francisco Bay‐Area clinics of Kaiser Permanente Northern California. Participants at the clinical site were 3–5 years of age and referred to the AJ Drexel Autism Institute for clinical evaluation from a community partner organization due to suggestion of developmental delays and suspicion of ASD. Families who indicated willingness to participate in research were offered enrollment by clinicians performing the evaluation. Participants at the general population site were recruited from the San Jose and Oakland Kaiser Permanente clinics as part of an ongoing research project participating in the Environmental Influences on Child Health Outcomes (ECHO) Program (Gillman & Blaisdell, 2018). Children attending the study visit were 4 years of age and approached based on their participation in ECHO, which is a large collaborative project seeking to examine a range of factors in association with child health across 5 key child outcome areas, including neurodevelopment, pre‐ peri‐ and neonatal, obesity, asthma/airways, and positive health. Enrollment took place between January 2019 and February 2021.

### Ethical considerations

All participants consented to participation and utilization of information for research purposes. This study was reviewed and approved by the Institutional Review Board (IRB) of Drexel University.

### SRS administration and scoring

#### Full and short SRS

Following approval from the publisher to modify administrations, short SRS questionnaires were created including only the 16 items (see Appendix S1), listed in order from the original SRS. The official published full‐length (65‐item; hereafter referred to simply as the ‘full SRS’) school‐aged forms were administered for children aged 4 and older, and preschool forms for children <4 years. Preschool and school age forms are very similar; 10 items in the preschool SRS include wording modifications and age‐appropriate examples, while four items include more minor simplifications, to align with developmental relevance. Raw total SRS scores for the short and full SRS were calculated following publisher guidelines, by summing item scores following reverse coding of select items (Constantino & Gruber, 2012).

#### CAT‐SRS

The CAT was developed as an adaptive algorithm to tailor SRS questions administered based on an individual's previous responses. Details of the CAT‐SRS development have been described elsewhere (Kaat et al., manuscript) and are also summarized in Appendix S2. Briefly, existing SRS data from 11 cohorts participating in ECHO (Lyall et al., 2021) as well as other large, general population studies (Mulligan et al., 2014), were used in initial IRT model fitting to estimate latent score distributions and test item parameters by form (preschool/school‐age) and sex assigned at birth (when differential item functioning was suspected). A multiple‐group IRT model was run with the mirt and mirtCAT packages in R to estimate item parameters and later to administer the CAT‐SRS (Chalmers, 2012). Simulated CAT models evaluated statistical efficiency under various stopping rules. The final CAT algorithm required a minimum of 10 items (investigator‐defined minimum for yielding a quantitative score), and stopped when both the minimum number of items was reached and either empirical reliability was >0.90 (SE < 0.3) or score equilibrium was reached (Babcock & Weiss, 2012), as indexed by the change in expected a posteriori (EAP) score estimates between two consecutive items (Thissen et al., 1995) (i.e., |ΔEAP| < 0.05).

EAP scores were estimated with a diffuse prior and from the administered items on the CAT‐SRS. Then, each EAP score was converted to an expected test score (as if the individual completed all SRS items) (Chalmers, 2012), which we will refer to as ‘CAT‐predicted score’ or simply ‘CAT score’ hereafter. This IRT‐based scaling method places the estimated latent score on the same scale as the observed score, allowing comparisons that would not have otherwise been possible with the CAT‐SRS.

### Data collection procedures

A block randomization scheme was used to enroll participants to receive one of the three SRS versions: the full 65‐item SRS, the short 16‐item SRS, or CAT‐SRS. For the first 11 months of the study while the CAT was under development, participants were assigned to the full or short SRS. Randomization to the CAT version of the SRS began in December 2019 and continued until the end of the recruitment period. Recruitment and administration of the study measures took place in person, with short and full SRS administered as paper questionnaires, until March 2020. Participation continued remotely, due to the COVID‐19 pandemic, until the end of the study. Remote CAT administration took place through Zoom, with research staff sharing SRS items on the screen for parents to read and indicate their response verbally. At the Kaiser general population site, participants administered the short and CAT‐SRS were given ‘remaining’ SRS items not included in these shortened administrations, after they completed the shortened versions, given the preference to obtain full SRS data in the larger ECHO study. (For example, if a CAT‐SRS administered items 1–10, the participant would then be given items 11–65).

Additional information collected included basic demographic characteristics and diagnostic status. Participants at the clinical site were asked to complete a brief demographic questionnaire assessing primary caregiver/respondent information on race/ethnicity and education. Information on these characteristics was obtained from medical records at the Kaiser general population site. Autism spectrum disorder diagnosis at both sites was determined based on clinical evaluations by trained professionals, information collected during evaluations including the Autism Diagnostic Observation Schedule (Second Edition) (Lord et al., 2012), and meeting DSM‐5 (clinical site) or ICD‐9 (general population site) criteria. Information on presence or absence of other neurodevelopmental, medical, and psychiatric diagnoses (including seizure disorder, motor delay, anxiety, intellectual disability, speech/language delay and other psychiatric and developmental disorders including attention deficit hyperactivity disorder) was collected via records (ICD‐9 codes or clinical record indication).

### Statistical analysis

Descriptive statistics of baseline characteristics and SRS scores were examined overall and by site. To facilitate the comparison of the three different SRS versions, which yield differing total score ranges (full:0–195; short:0–48; CAT: variable by participant's total items administered, but potential range 0–195), primary analyses used equipercentile equating to scale short scores (Schalet et al., 2021) (Appendix S3), and predicted full total scores (as described above) for CAT‐SRS scores. Comparisons relying on other scaling methods (including percent of maximum possible) were examined in secondary analyses. Cohen's d was calculated to compare differences in means across factors.

In order to address our first primary goal of comparing score distributions of the SRS versions, in addition to comparison of descriptive statistics across measures, distributional properties of the three SRS versions were compared visually using density plots. Plots were created separately by site, ASD status, and sex. Comparisons by form (preschool vs. school age) were also conducted for the clinical site only (given school‐aged forms were used for nearly all individuals at the general population site). In addition, plots were created for within‐individual comparisons of short and CAT versus full scores from the relevant groups at the Kaiser site. Bland‐Altman plots were also used to visualize agreement between the full SRS and shortened (16‐item and CAT) administrations within this group.

To assess predictive ability to address our second primary aim, SRS scores from each version were compared to ASD diagnoses using Receiver Operating Characteristic (ROC) analyses for the entire sample. ROC analyses were also conducted stratified by sex. Given the small number of ASD cases from the general population site, and small number of non‐cases at the clinical site, these analyses were not conducted stratified by site. Chi‐squared tests were used to statistically compare areas under the ROC curves across groups. As a secondary comparison and exploratory analysis to assess specificity to ASD diagnosis, we also conducted ROC analyses assessing SRS scores ability to predict other, non‐ASD diagnosis as indicated in medical and clinical assessments (further described in Figure S7). Finally, we also examined participant characteristics and scores according to collections that occurred pre‐ and during COVID.

## RESULTS

The average child age was similar across sites, but slightly younger at the clinical site (mean 3.5 vs. 4.1 years at clinical vs. general population site respectively). Children from the clinical site were more likely than those from the general population site (as expected) to have an ASD diagnosis (84% vs. 2%), and to be male (72% vs. 34%) (Table 1). Following age differences, nearly all participants at the general population site were administered the school‐age form, while the majority at the clinical site were administered the preschool form. Though numbers were small in several groups, maternal race, ethnicity, and education also differed across sites, with a greater proportion of Black mothers at the clinical site, more Hispanic mothers at the general population site, and lower educational attainment at the clinical site. The vast majority of respondents at both sites were mothers. Demographic trends remained largely consistent when broken down by administration type within each site, and groups were broadly comparable by demographic factors within site (Tables S1A and S1B).

**TABLE 1:** Basic characteristics of study participants by site (*n* = 355)

Characteristic^a^	Clinical site	General population site
	*n* = 154	*n* = 201
Child's age (yrs) mean (SD)	3.5 (0.7)	4.1 (0.2)
Child's sex		
Male	112 (73%)	68 (34%)
Female	42 (27%)	133 (66%)^b^
Informant's relationship to child		
Mother	127 (82%)	201 (100%)
Other guardian/caregiver	27 (18%)	
Mother's race		
White or caucasian	34 (22%)	129 (64%)
Black or African‐American	80 (52%)	7 (3%)
Asian	11 (7%)	22 (11%)
Biracial or multiracial	6 (4%)	17 (8%)
Hawaiian Or Pacific Islander	1 (1%)	2 (1%)
Other	5 (3%)	2 (1%)
Unknown	17 (11%)	22 (11%)
Mother's ethnicity		
Hispanic	31 (20%)	59 (29%)
Non‐Hispanic	106 (69%)	138 (69%)
Unknown	17 (11%)	4 (2%)
Mother's education		
HS degree or less	49 (32%)	7 (3%)
Some College/trade school	44 (29%)	59 (29%)
College degree	33 (21%)	78 (39%)
Post‐college degree	14 (9%)	50 (25%)
Unknown	14 (9%)	7 (3%)
ASD diagnosis		
Yes	130 (84%)	4 (2%)
No	24 (16%)^c^	197 (98%)
SRS form		
Preschool	97 (63%)	1 (0.5%)
School‐age	57 (37%)	200 (99.5%)
SRS version/administration method		
Full 65‐item SRS	46 (30%)	67 (33%)
Short 16‐item SRS	58 (38%)	67 (33%)
CAT SRS	50 (32%)	67 (33%)

Abbreviations: ASD, Autism Spectrum Disorder; SRS, Social Responsiveness Scale.

^a^ N (%) shown unless indicated.

^b^ Sex ratio was roughly even in full and short groups, but skewed mainly female in CAT group.

^c^ 1 individual had inconclusive diagnostic status following evaluation and was included in the ‘no’ category.

The most commonly administered items within the CAT‐SRS by site are shown in Table S2. At both sites, the CAT version tended to administer 14–15 items (range 10–33) before stopping.

Mean scores were substantially higher in the clinical group overall (the majority of whom received an ASD diagnosis) as compared to the general population group for all three versions of the SRS (Table 2). Differences between sites by version ranged from approximately 50 to 80 points (Cohen's d of 1.8 for the full, 2.1 for the short, and 3.2 for the CAT). Comparing administration methods, shortened version scores were generally higher in those from the clinical site (short: 7.8 points higher; CAT: 23.0 points higher), and lower or comparable in those from the general population site (short: 2.4 points higher; CAT: 7.3 points lower) than full SRS scores. Scores in females were on average lower than in males by approximately 3–15 points across versions, with the exception of short scores from females from the general population (which were an average of 4 points higher). At both sites, the children of mothers identifying as Hispanic tended to have higher scores than mothers identifying as Non‐Hispanic, while scores in the children of mothers identifying as Black were lower in the general population group (but not uniformly across versions in the clinical group), though participant numbers in some of these groups were small and therefore should be interpreted with caution.

**TABLE 2:** Descriptive statistics of Social Responsiveness Scale (SRS) scores by administration method (full 65‐item, short 16‐item, or computer adaptive testing (CAT)) and study site (Constantino & Todd, 2003)

	Clinical site				General population site			
		Full (*n* = 46)	Short^b^ (*n* = 58)	CAT^c^ (*n* = 50)		Full (*n* = 67)	Short^b^ (*n* = 67)	CAT^c^ (*n* = 67)
	*n*	Median (IQR)			*n*	Median (IQR)		
Overall	154	66.50 (54.00)	87.10 (37.30)	104.10 (36.69)	201	27.00 (18.00)	23.90 (19.80)	16.62 (13.20)
ASD diagnosis^d^								
Yes	130	71.00 (49.00)	88.45 (31.50)	105.94 (35.10)	4	117.00 (6.00)	106.80 (66.40)	‐
No	23	57.00 (42.00)	67.80 (76.80)	72.32 (84.81)	197	25.00 (17.00)	23.90 (19.80)	16.62 (13.20)
Ethnicity								
Non‐Hispanic	106	65.00 (57.00)	79.10 (47.10)	104.10 (36.70)	138	24.00 (18.00)	23.90 (19.80)	18.09 (11.12)
Hispanic	31	101.50 (30.00)	95.50 (57.10)	103.92 (52.72)	59	27.00 (16.00)	37.75 (15.80)	15.51 (11.76)
Unknown ethnicity	17	64.00 (46.00)	89.80 (46.10)	105.51 (33.52)	4		12.80 (14.20)	18.94 (16.09)
Race^e^								
Black or African American	80	65.00 (60.00)	80.45 (37.50)	106.43 (34.76)	7	15.50 (1.00)	21.90 (4.00)	16.45 (10.53)
Asian	11	59.50 (26.00)	89.05 (39.75)	122.43	22	35.00 (30.00)	23.90 (19.80)	23.18 (10.00)
White	34	65.50 (64.00)	95.50 (48.70)	102.89 (41.38)	129	24.00 (17.00)	27.90 (19.80)	15.76 (10.67)
Biracial or multiracial	6	77.50 (13.00)	85.65 (84.30)	79.93 (15.21)	17	27.00 (5.00)	19.90 (16.10)	17.30 (11.79)
Unknown race	17	110.00 (12.00)	92.65 (41.00)	104.52 (8.14)	22	38.00 (31.00)	29.80 (43.85)	16.23 (22.79)
By child's sex								
Males	112	67.00 (60.00)	81.80 (34.40)	109.24 (36.12)	68	29.00 (23.00)	23.90 (19.80)	18.69 (25.97)
Females	42	64.00 (48.00)	89.80 (84.00)	101.41 (31.63)	133	25.00 (10.50)	27.90 (19.80)	16.62 (12.56)
By form								
Preschool	97	69.50 (57.00)	84.45 (43.70)	102.90 (43.90)	1			^a^
School age	57	66.50 (50.50)	92.65 (55.10)	109.78 (31.06)	200	27.00 (18.00)	23.90 (19.80)	16.60 (12.56)

^a^ Indicates data suppressed due to only 1 participant for this group.

^b^ Short scores scaled according to equipercentile equating method.

^c^ CAT‐predicted scores.

^d^ Diagnosis for one participant was inconclusive and not included in ASD comparisons.

^e^ Groups with 5 or fewer participants in racial categories across both sites (Hawaiian/Pacific Islander and Other Race) not shown due to small samples.

**FIGURE 1:**
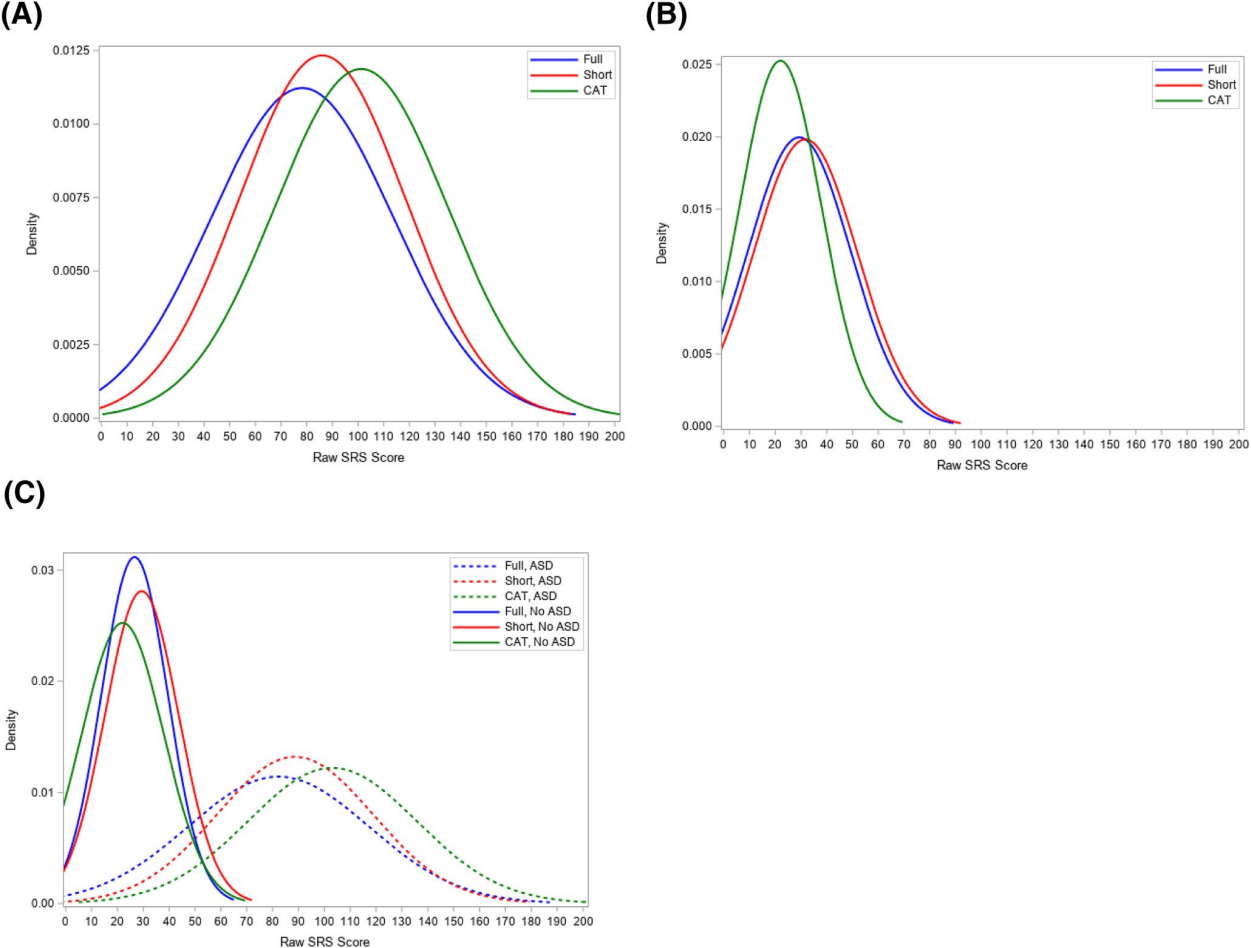
Distributions of Social Responsiveness Scale (SRS) scores by study site. (A) Clinical Site (B) General Population Site (C) By Site and Autism spectrum disorder (ASD) status. Distributional plots using normal density function for full 65‐item raw SRS scores (blue lines), short 16‐item scores (red lines) scaled to full raw scores using equipercentile equating, and CAT‐predicted total raw scores (calculated as summarized in the text) (green lines). (A) Clinical site, including 154 participants referred for autism diagnostic evaluation (with 46, 58, and 50 participants in each of the full, short, and computer adaptive testing (CAT) groups, respectively) and (B) 201 participants from the general population site (with 67 participants in each of the full, short, and CAT groups) (B). Plot (C) shows scores across administration methods separated by child's ASD diagnostic status, with ASD case scores shown as dotted lines and non‐cases as solid lines. Individuals referred for clinical evaluation but not diagnosed with ASD were not included, though the density plot including these individuals was similar.

Overall, SRS score distributions from the three versions were highly overlapping at each site (Figure 1A,B). Short scores were shifted slightly right and fell within 0.1 and 0.2 SD of full scores at the general population and clinical sites respectively, while CAT‐predicted scores were shifted slightly right of full scores (within 0.7 SD) at the clinical site and slightly left (within 0.4 SD) at the general population site (Table 2; Figure 1A,B). Score separation between ASD cases and non‐cases was high across all versions, from approximately a 2 standard deviation difference for full and short scores, to approximately a 3 SD difference for CAT scores (Figure 1C; Table S3). Scores in males were shifted slightly to the right as compared to females for all versions and sites (Figure S1), with the exception of female short scores in the general population group (which were highly overlapping but modestly shifted to the right of full male scores by ∼0.2 SD). Examining school‐age versus preschool forms, cross‐version patterns were similar to those observed overall, though preschool scores were shifted left by approximately 0.2–0.3 SD relative to school‐aged scores for all versions but the full (which showed greatest overlap; Figure S2).

Comparing short and CAT scores to corresponding full scores obtained from the same individuals at the general population site, agreement was generally high, though both short and CAT scores tended to be slightly lower than full scores (by a mean difference of approximately two points for short and 9 points for the CAT) (Table S4; Figure S3). Bland‐Altman plots also demonstrated overall high within‐subject agreement between the full and shortened scores (Figure S4); over 95% of observations fell within 2 standard deviations of no mean difference.

**FIGURE 2:**
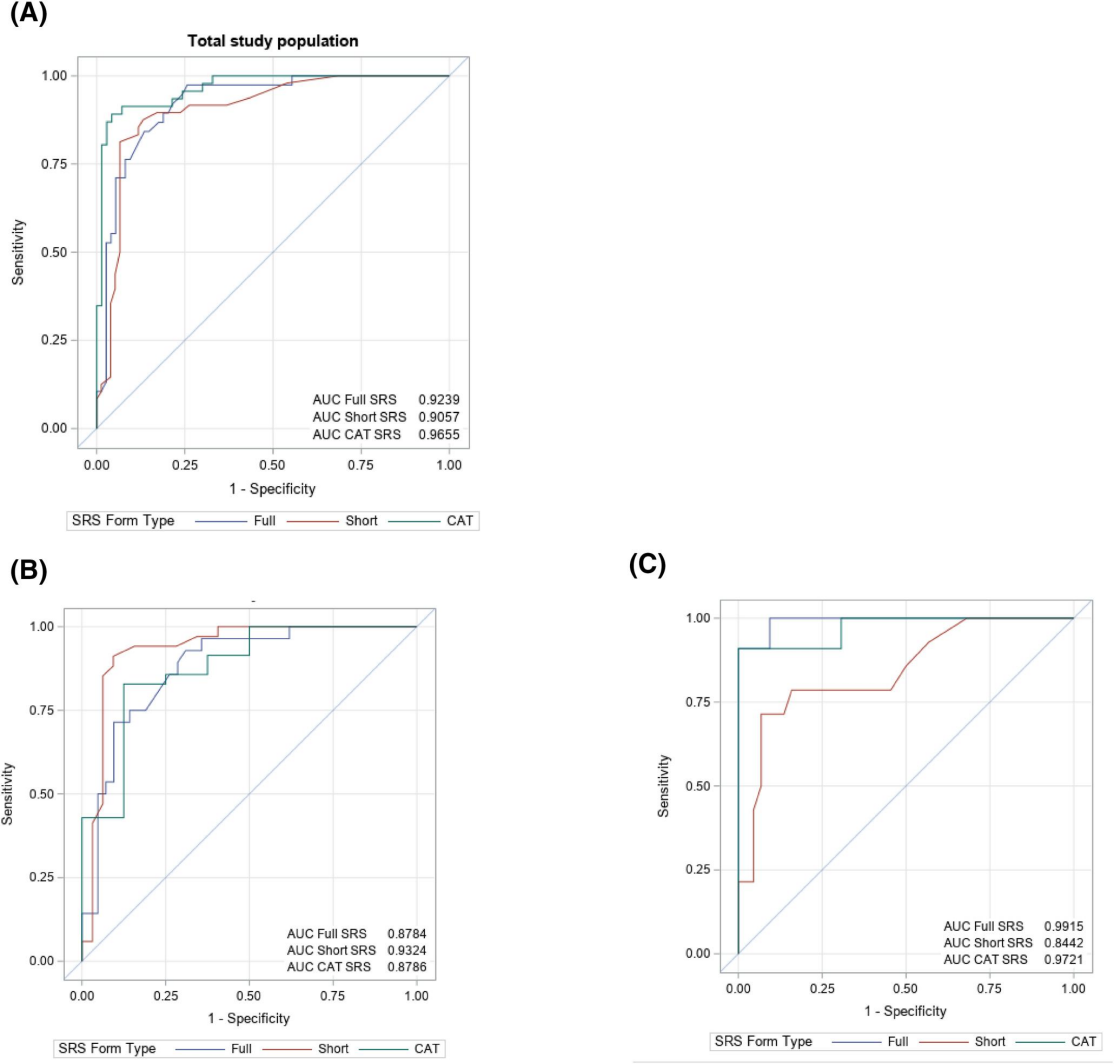
Receiver Operating Characteristic (ROC) curve predicting Autism spectrum disorder (ASD) status by Social Responsiveness Scale (SRS) version in (A) the total study population, (*n* = 354, including 134 ASD cases) (B) male children only, (*n* = 180, including 98 ASD cases) and (C) female children, (*n* = 174, including 36 ASD cases). As for distributional plots, full 65‐item SRS scores are shown in blue, short 16‐item scores (scaled per equipercentile equating) in red, and computer adaptive testing (CAT) scores (predicted raw total scores) in green. One individual from the CAT group is not included due to a missing CAT‐predicted score. No significant differences between AUC values for different SRS versions were observed in the total study population or male comparisons, but within females, there was a significant difference between the full AUC value (0.99) and the short value (0.84; *p* = 0.02)**.**

ROC analyses for the total study population suggested nearly identical performance of full, short, and CAT SRS scores in predicting ASD diagnoses (Figure 2A; AUC values of 0.92, 0.91, and 0.97, respectively). When stratified by sex, performance across measures remained high, though the short version performed slightly better in boys (Figure 2B; AUC values of 0.88, 0.93, and 0.88 for full, short, and CAT respectively), while the full version performed best in girls (Figure 2C; AUC values of 0.99, 0.85, and 0.97 for full, short, and CAT, respectively). Among school‐aged participants, the full, short, and CAT SRS demonstrated almost identical performance in predictive ability (Figure S5a; values were lower for the preschool form, S5b). AUC values as well as sensitivity, specificity, and cut‐off scores are shown in Table S5.

In secondary analyses, similar descriptive statistics and distributional relationships across administration methods were observed using the other scaling method tested (POMP‐scaled scores, Table S6; Figure S6). Exploratory analyses of prediction of other, non‐ASD neurodevelopmental or psychiatric outcomes noted in records (excluding those with ASD; *n* = 46, the majority of which were non‐ASD developmental delays or speech/language disorders; a complete list is provided in Figure S7) was similar across SRS versions (Figure S7; AUC values of 0.62, 0.77, and 0.67, for full, short, and CAT respectively), but notably lower than values obtained for prediction of ASD diagnosis (Figure 2A). Approximately half of the clinical CAT group, and over 80% of the general population group, had CAT‐SRS scores collected during COVID. Participant characteristics were similar pre‐ and during COVID (Table S8), with the exception of more girls in the latter at the general population site, and scores were also comparable across these time frames (Table S9).

## DISCUSSION

Results from this validation study suggest the broad utility of the two shortened versions of the SRS tested here in young children. Across the full, short, and CAT‐SRS, we observed overall similarity in distributions, suggesting comparability in shortened versions for use as quantitative trait measures. We also observed similarity in prediction of ASD, especially in males, which supports comparability for screening purposes. Several considerations, including sex differences in performance for shortened versions, as well as recommendations for studies using the SRS and areas for further development, are detailed below.

We were interested in whether shortening the SRS had a substantial impact on performance both as a quantitative trait measure and for screening purposes ‐ two primary uses of the SRS in research studies. We previously observed evidence of comparability of 16‐item scores and 65‐item scores using existing data drawn from 11 ECHO cohorts, representing over 2400 participants (Lyall et al., 2021). The work here serves to extend these findings by basing comparisons on actual administrations of the shortened measure as a stand‐alone questionnaire, rather than as drawn from full 65‐item questionnaires. A large number of prior studies have supported the validity of the full SRS in capturing ASD‐related traits (Constantino & Gruber, 2012). The SRS has been validated against a ‘gold standard’ for diagnosis, the Autism Diagnostic Interview‐Revised (ADI‐R), with strong results (*r* = 0.7 for SRS scores and ADI‐R algorithm scores for DSM‐IV criteria) (Constantino et al., 2003, 2009). Prior work has also supported high internal validity, reliability, reproducibility, and score stability (Constantino et al., 2003, 2009; Constantino & Gruber, 2012), and strong performance in both the general population (Constantino & Todd, 2003) and in ASD families (Constantino et al., 2006).

However, some have raised concerns about specificity of SRS scores to ASD. Our exploratory analysis of prediction of other, non‐ASD developmental and psychiatric conditions demonstrated lower values than those obtained for prediction of ASD (with AUC values falling in the poor to low acceptable range for other conditions), in contrast to AUC values falling in the excellent to outstanding range for ASD prediction. This comparison adds to prior work supporting that scores capture traits that are consistent with ASD diagnosis. These analyses also suggested better prediction of other non‐ASD diagnoses with the shortened versions than the full SRS (as evidenced by higher AUC values for the CAT and short SRS than the full SRS in predicting other diagnoses), implying that shortened versions may compromise the separation from other diagnoses. Although our work was not optimally designed to address information on co‐occurring conditions given several limitations, including the age range of our sample, this suggestion is consistent with early SRS development (Constantino et al., 2000), and shortened comparisons for another continuous autism trait measure, the Autism Screening Questionnaire (Berument et al., 1999). On the other hand, development of an abbreviated version of the Social Communication Questionnaire suggested increased specificity in clinical samples (as did the short SRS development in ASD‐skewed samples) (Sturm et al., 2017) but better performance of the full in general population (Marvin et al., 2017). We also observed reduced separation between ASD case and non‐case scores in the clinically‐ascertained sample relative to the general population sample. All children at the clinical site were referred for evaluation due to suspicion of ASD, and therefore expression of the ASD‐related phenotype; this sampling will necessarily skew the distribution observed in the clinical group that did not ultimately receive an ASD diagnosis. Given the high co‐occurrence in categorically‐defined conditions (Hossain et al., 2020) (for example, estimates of ∼30–70% of children with ASD also having ADHD) (Taurines et al., 2012), the overlap in continuous score distributions of phenotypic traits and reduction in separation of mean scores follows as an expected consequence of this categorical overlap. Furthermore, evidence suggests a high degree of etiologic overlap across psychiatric and developmental diagnostic categories (Eaton et al., 2001; Kelleher & Corvin, 2015) (like ASD and ADHD) (Taurines et al., 2012), an observation that also lends itself to the expectation of overlap in latent traits.

While the overall landscape of results suggests comparable utility of these shortened SRS measures to the full SRS, there are a few key caveats or considerations worth highlighting. The first, and most notable, is the observation of differences in ASD prediction for females that suggests that more questions, or potentially, targeted questions, may be needed to accurately assess diagnostic status in female children. This conclusion is drawn from the observed differences in predictive ability in full versus short SRS scores (but not full vs. CAT scores) for females only. Though we had relatively small numbers in our study to address sex differences, and cannot rule out potential chance variation in our findings, these potential sex differences are worth consideration. While sex differences of ∼4:1 male:female ratio are consistently reported, there is also evidence that at least some proportion of diagnostic differences by sex could be due to under‐recognition and misdiagnosis in females (Halladay et al., 2015; Whitlock et al., 2020). Such delays or misses in turn may have negative impacts on other emotional, academic, and lifecourse outcomes (Atherton et al., 2021). Though debated (Fombonne, 2020), there have also been suggestions in the literature of differences in presentation and ‘camouflaging’ of ASD symptoms and social deficits in females (Corbett et al., 2021; Green et al., 2019), which could relate to our finding of possible compromises in assessment of phenotype in females with the short version. The second major consideration is that there are restraints on the ability to examine subscales assessing more specific aspects of the ASD‐related phenotype with the shortened measures, a compromise that comes with item reduction (see also Table S7). A third consideration is some evidence for underestimation of full scores with shortened versions (particularly the CAT in the general population sample), suggesting potential for modest underestimation of the phenotype. And the final points are that, while we may have reasonable data to assume comparability with full measure metrics (Lyall et al., 2021; Nguyen et al., 2008), neither heritability nor test‐re‐test reliability of these shortened versions have been directly assessed. Therefore, studies seeking to utilize the SRS need to carefully weigh these factors and align version choice with the goals of their study.

Key areas for further work include investigation of phenotype measurement and accuracy of shortened measures in females, as well as determining whether the difference for females in predictive ability between short and full SRS versions persists (or increases) in later ages. Given variability across racial and ethnic groups (both in our project as well as in other studies (Constantino & Gruber, 2012)), defining whether item‐level differences exist by racial and ethnic groups, some of whom may experience underdiagnosis issues that parallel those of females for ASD, also represents a research need, particularly given limited diversity in this study.

This is the first study to directly assess the validity of these shortened versions of one of the most widely‐used quantitative measures of ASD‐related phenotype. Key strengths include examination in both a clinical and a general population setting and incorporation of a novel, adaptive computer administration mode. However, several limitations should also be noted. Our sample size was relatively small, limiting our ability to compare subgroups. Furthermore, while we reached our enrollment target at the general population site, enrollment was impacted by the COVID‐19 pandemic, particularly for families of children with a known or suspected developmental condition. Calculations suggest we still had reasonable power (>70% to effect sizes of *d* = 0.2, smaller than observed here) even with the approximate 12% reduction in overall target sample size. We also cannot rule out potential impacts of the COVID‐19 pandemic, which may have caused CAT‐SRS scores to differ from the other administrations due to our block randomization scheme. However, scores collected pre‐COVID and during COVID were similar. Furthermore, findings from within individual comparisons at the general population site were consistent with those seen across‐groups, suggesting any potential impacts of COVID on our study did drive differences observed across administration modes. Choice of scaling for shortened scores may impact comparative performance, though we tested two scaling methods that yielded comparable findings. In addition, prior work comparing scaling methods, though noting greater sensitivity of equipercentile equating for sparse and variable data, has suggested similarity in calibration methods (Schalet et al., 2021). Finally, although data supports measurement invariance of SRS scores across age (Duku et al., 2013; Frazier et al., 2014), continued assessment of measurement invariance across preschool and school ages is needed. Children included in this study were young, and while the high correlation between shortened and full scores observed here suggests reasonable confidence in translation of full score properties like stability of SRS scores over time (Constantino et al., 2009; Haraguchi et al., 2018; Stickley et al., 2017), we cannot rule out potential differences in older ages. We also had limited ability to compare to other diagnoses, particularly those that may arise at later ages. Future work should in particular consider measurement of shortened scores in adults, and also as compared to self‐report, as may help to learn more about self‐identified autistic traits.

Quantitative trait measures like the SRS offer the ability to consider not only variability in phenotype within autistic individuals, but also, to capture subclinical features, and traits across the population. Our study adds to existing literature by suggesting broad comparability across the full and shortened versions here and provides evidence for improvements in efficiency without substantial reductions in overall construct measurement (with the above‐noted caveats). The availability of shortened SRS versions presents opportunities for collection of ASD‐related phenotype data in settings where participant burden or feasibility considerations may have otherwise prohibited such measurement. Wider‐scale collection of data on these traits, in turn, can complement ASD diagnostic data to ultimately enrich our understanding of the phenotype.

## AUTHOR CONTRIBUTIONS

Study conceptualization and funding (Kristen Lyall and Craig J. Newschaffer); primary data collection and coordination (James Connell, John N. Constantino, Lisa A. Croen, Bridget Toroni, Kate Piselli, Brigid Garvin), primary manuscript drafting (Kristen Lyall, Bridget Toroni, and Juliette Rando), statistical analyses (Aaron J. Kaat, Juliette Rando, Tobechukwu Ezeh), manuscript editing (all authors), study guidance (Kristen Lyall, John N. Constantino, Craig J. Newschaffer, Aaron J. Kaat, Lisa A. Croen), final manuscript approval (all authors).

## CONFLICTS OF INTEREST

John N. Constantino receives royalties from Western Psychological Services for the commercial distribution of the Social Responsiveness Scale‐2. The remaining authors have declared that they have no competing or potential conflicts of interest.

## ETHICAL CONSIDERATIONS

All participants consented to participation and utilization of information for research purposes. This study was reviewed and approved by the Institutional Review Board (IRB) of Drexel University.

## Supporting information

Supporting Information S1

## Data Availability

The data that support the findings of this study are available on request from the corresponding author. The data are not publicly available due to privacy or ethical restrictions.
