# Supplementary material for: Examining shortened versions of the Social Responsiveness Scale for use in autism spectrum disorder prediction and as a quantitative trait measure: Results from a validation study of 3–5 year old children
Source: JCPP Adv. 2022 Oct 5;2(4):e12106. doi: 10.1002/jcv2.12106 (PMC9890399; doi:10.1002/jcv2.12106)
Supplement: Supplementary file 1 — Supporting Information S1 [file JCV2-2-e12106-s001.docx]

**SUPPORTING INFORMATION**

**SUPPLEMENTARY TABLES AND FIGURES**

*Appendices provided at the end of this document.*

**Table S1a.** Basic characteristics of clinical site study participants by administration method (n=154)

| Variable (n % unless indicated) | SRS Version | | |
| --- | --- | --- | --- |
|  | Full | Short | CAT |
| Child’s Age |  |  |  |
| Mean (SD) | 3.5 (0.78) | 3.5 (0.71) | 3.4 (0.70) |
| Child’s Gender |  |  |  |
| Male | 35 (76.1) | 39 (67.2) | 38 (76.0) |
| Female | 11 (23.9) | 19 (32.8) | 12 (24.0) |
|  |  |  |  |
| Informant’s Rel. to Child |  |  |  |
| Mother | 33 (71.7) | 52 (89.7) | 42 (84.0) |
| Father | 11 (23.9) | 2 (3.4) | 4 (8.0) |
| Legal Guardian | 1 (2.2) |  |  |
| Other |  | 4 (6.9) | 2 (4.0) |
| Unknown | 1 (2.2) |  | 2 (4.0) |
| Mother’s Race |  |  |  |
| White or Caucasian | 10 (21.7) | 13 (22.4) | 11 (22.0) |
| Black or African-American | 23 (50.0) | 30 (51.72) | 27 (54.0) |
| Asian | 6 (13.0) | 4 (6.9) | 1 (2.0) |
| Biracial or Multiracial | 2 (4.4) | 2 (3.5) | 2 (4.0) |
| Hawaiian or Pacific Islander |  |  | 1 (2.0) |
| Other |  | 1 (1.7) | 4 (8.0) |
| Unknown | 5 (10.9) | 8 (13.8) | 4 (8.0) |
| Mother’s Ethnicity |  |  |  |
| Hispanic | 6 (13.0) | 15 (25.9) | 10 (20.0) |
| Non-Hispanic | 35 (76.1) | 35 (60.3) | 36 (72.0) |
| Unknown | 5 (10.9) | 8 (13.8) | 4 (8.0) |
| Mother’s Education |  |  |  |
| Less than High School or GED | 1 (2.2) | 4 (6.9) | 2 (4.0) |
| High School Diploma or GED | 13 (28.3) | 17 (29.3) | 12 (24.0) |
| Technical or Trade School | 2 (4.4) | 8 (13.8) | 4 (8.0) |
| Some College | 9 (19.6) | 8 (13.8) | 13 (26.0) |
| College Degree | 10 (21.7) | 10 (17.2) | 13 (26.0) |
| Post-College Degree | 4 (8.7) | 6 (10.3) | 4 (8.0) |
| Unknown | 7 (15.22) | 5 (8.62) | 2 (4.0) |
| ASD Diagnosis |  |  |  |
| Yes | 37 (80.4) | 46 (79.3) | 47 (94.0) |
| No | 9 (19.6) | 11 (19.0) | 3 (6.0) |
| Inconclusive |  | 1 (1.7) |  |
| Other neurodevelopment diagnosis^1^ |  |  |  |
| Yes | 4 (8.7) | 5 (8.6) | 2(4.0) |
| No | 42 (91.3) | 53(91.4) | 48 (96.0) |
| Form Version |  |  |  |
| Preschool | 30 (65.2) | 38 (65.5) | 29 (58.0) |
| School-age | 16 (34.8) | 20 (34.5) | 21 (42.0) |
|  |  |  |  |

^1^Other diagnosis includes the following conditions: ADHD, seizure disorder, motor delay, anxiety, intellectual disability, speech/language delay and other psychiatric and developmental disorders.

**Table S1b.** Basic characteristics of general population site study participants by administration method (n=201)

| Variable (n % unless indicated) | SRS Version | | |
| --- | --- | --- | --- |
|  | Full | Short | CAT |
| Child’s Age |  |  |  |
| Mean (SD) | 4.1 (0.26) | 4.0 (0.14) | 4.1 (0.24) |
| Child’s Gender |  |  |  |
| Male | 35 (52.2) | 27 (40.3) | 6 (9.0) |
| Female | 32 (47.8) | 40 (59.7) | 61 (91.0) |
| Informant’s Rel. to Child |  |  |  |
| Mother | 67 (100) | 67 (100) | 67 (100.0) |
| Mother’s Race |  |  |  |
| White or Caucasian | 50 (74.6) | 48 (71.6) | 31 (46.3) |
| Black or African American | 2 (3.0) | 2 (3.0) | 3 (4.5) |
| Asian | 2 (3.0) | 7 (10.4) | 13 (19.4) |
| Biracial or Multiracial | 5 (7.5) | 3 (4.5) | 9 (13.4) |
| Hawaiian or Pacific Islander | 1 (1.5) | 1 (1.5) |  |
| Other |  | 2 (3.0) |  |
| Unknown | 7 (10.4) | 4 (6.0) | 11 (16.4) |
| Mother’s Ethnicity |  |  |  |
| Hispanic | 25 (37.3) | 14 (20.9) | 20 (29.9) |
| Non-Hispanic | 42 (62.7) | 51 (76.1) | 45 (67.2) |
| Unknown |  | 2 (3.0) | 2 (3.0) |
| Mother’s Education |  |  |  |
| High School Diploma or GED | 4 (6.0) | 2 (3.0) | 1 (1.4) |
| Technical or Trade School |  | 1 (1.5) | 2 (3.0) |
| Some College | 15 (22.4) | 21 (31.3) | 20 (30.0) |
| College Degree | 34 (50.8) | 23 (34.3) | 21 (31.3) |
| Post-College Degree | 14 (20.9) | 17 (25.4) | 19 (28.4) |
| Unknown |  | 3 (4.5) | 4 (6.0) |
| ASD Diagnosis |  |  |  |
| Yes | 2 (3.0) | 2 (3.0) |  |
| No | 65 (97.0) | 65 (97.0) | 67 (100.0) |
| Other neurodevelopment diagnosis^1^ |  |  |  |
| Yes | 18 (26.9) | 11 (16.4) | 11 (16.4) |
| No | 49 (73.1) | 56 (83.6) | 56 (83.6) |
| Form Version |  |  |  |
| Preschool |  |  | 1 (1.5) |
| School-age | 67 (100.0) | 67 (100.0) | 66 (98.5) |

^1^Other diagnosis includes the following conditions: ADHD, seizure disorder, motor delay, anxiety, intellectual disability, speech/language delay and other psychiatric and developmental disorders.

**Table S2.** Most commonly administered items within the CAT-SRS by site.

Table shows the ten most commonly administered items at each site within the CAT-SRS. Color coding is used to differentiate subscales. Item content has been summarized with permission from the publisher; actual questions are not reproduced as the content is copyrighted. The minimum number of items administered within the CAT-SRS was set to 10 for both sites. The average number of items administered was 14 at the clinical site and 15 at the general population site (with median, mode, and range of 12, 10 and 10-33 at the clinical site and 16, 16, and 10-32 at the general population site, respectively).

**Table** **S3**. SRS scores by SRS administration method and ASD status for total study population (both sites combined).

|  | **Total Study Population** | | | | |
| --- | --- | --- | --- | --- | --- |
|  |  | Full  (n=113) | Short^1^  (n=124) | CAT^2^  (n=117) | |
|  | n | Mean (std) | | | |
| ASD Diagnosis^3^  Yes  No | 134  220 | 83.46 (34.63)  31.19 (21.29) | 89.36 (30.57)  36.68 (26.34) | | 103.06 (32.69)  24.21 (19.98) |

^1^Short scores scaled according to equipercentile equating method. ^2^CAT-predicted scores; one individual is not included due to a missing CAT-predicted score. ^3^Diagnosis for one participant was inconclusive and not included in ASD comparisons.

**Figure S1.** Distributional plots by sex and administration method for clinical (a) and general population (b) sites.

1. Clinical site full, short, and CAT-SRS scores by sex


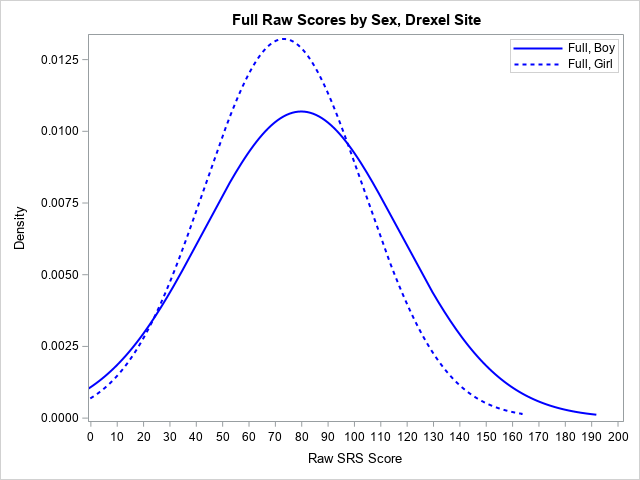

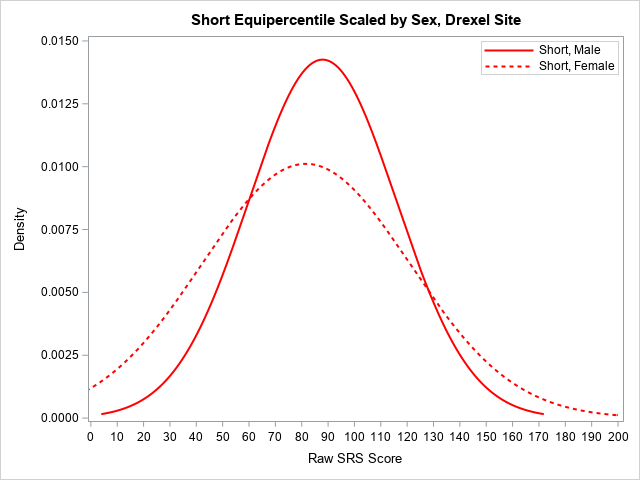

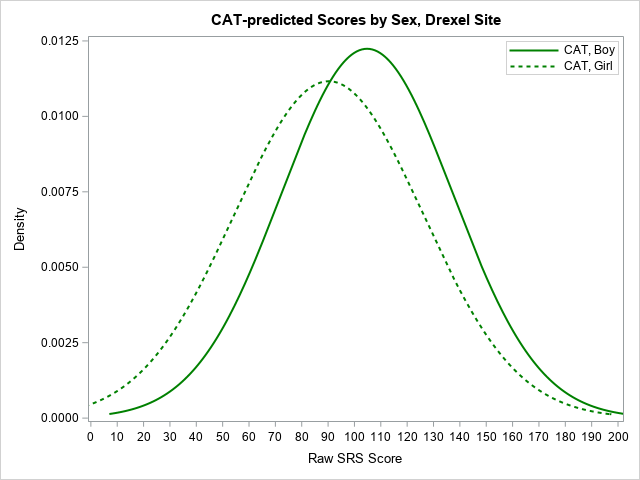


1. General population site full, short, and CAT-SRS scores by sex


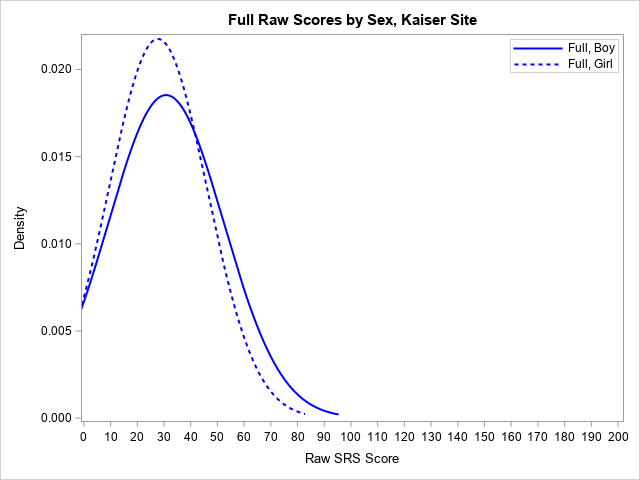

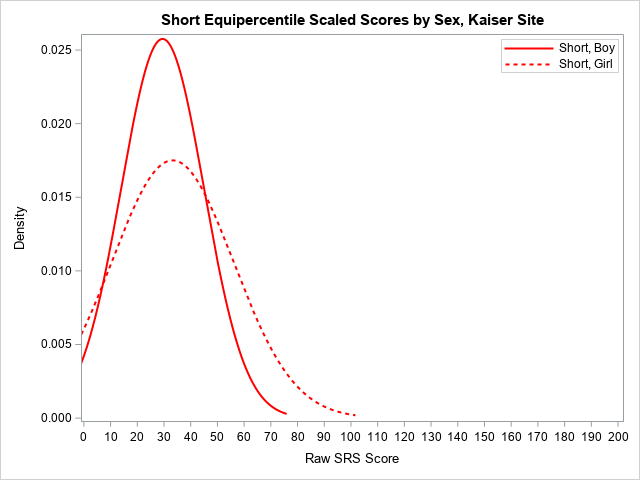

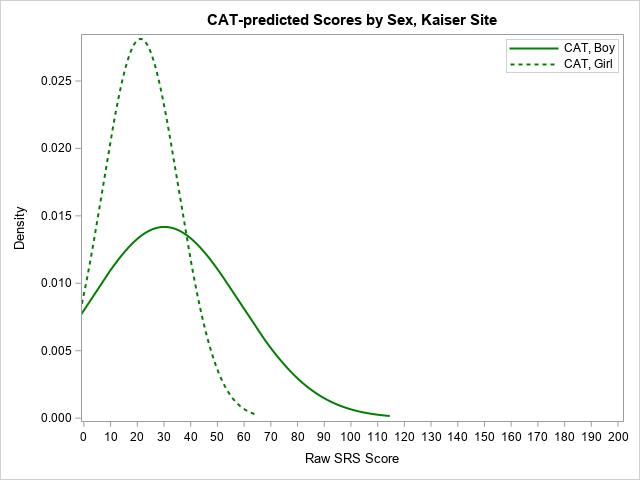


*Figure legend:* Distributional plots using normal density function for full 65-item raw SRS scores (blue lines), short 16-item scores (red lines) scaled to full raw scores using equipercentile equating, and CAT-predicted total raw scores (calculated as summarized in the text) (green lines). One individual from the CAT group is not included due to a missing CAT-predicted score.

**Figure S2.** Distributional plots by SRS version and administration method for the Drexel site.


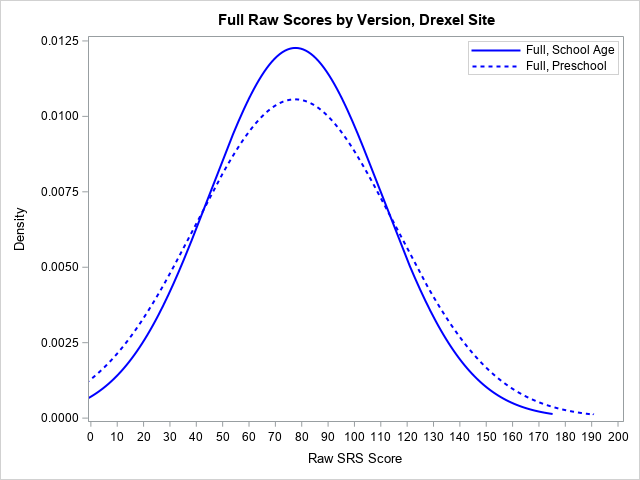

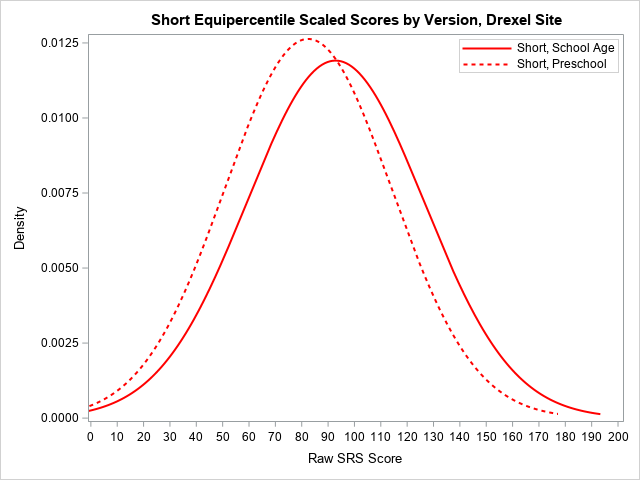

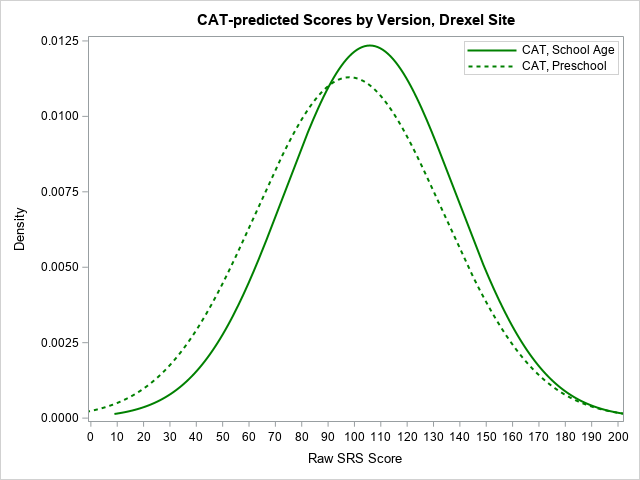


*Figure legend:* Distributional plots using normal density function for full 65-item raw SRS scores (blue lines), short 16-item scores (red lines) scaled to full raw scores using equipercentile equating, and CAT-predicted total raw scores (calculated as summarized in the text) (green lines). (N*ote: Preschool vs school aged score comparisons cannot be performed for the Kaiser site due to the small number (n=1) of preschool administrations.)* One individual from the CAT group is not included due to a missing CAT-predicted score.

**Table S4**. Within-subjects comparisons of full 65-item and CAT SRS scores for CAT participants and full 65-item and short 16-item SRS scores for short form participants at the general population site

|  | Short Group | | |  | CAT group | |
| --- | --- | --- | --- | --- | --- | --- |
|  |  | Full Score  (65-item)  (n=67) | Short Score^2^  (n=67) |  | Full Score  (65-Item)  (n=67) | CAT-predicted Score^1^  (n=67) |
|  | n | Mean (std) | | n | Mean (std) | |
| Overall | 67 | 34.00 (19.45**)** | 31.71 (20.12) | 67 | 31.30 (12.18) | 22.02 (15.80) |
| Among ASD cases^1^ | 2 | 103.00 (43.84) | 106.80 (46.95) | 0 | - | - |
| Among those with other neuro/psych disorder | 11 | 44.73 (20.04) | 41.64 (17.40) | 11 | 32.55 (9.37) | 23.54 (11.52) |
| By race/ethnicity  Non-Hispanic  Hispanic  Black or African American  Asian  White  Biracial or Multiracial | 51  14  2  7  48  3 | 33.33 (20.87)  37.29 (14.92)  23.50 (3.54)  37.14 (18.51)  34.00 (20.50)  23.33 (9.61) | 31.55 (21.20)  34.96 (15.92)  21.90 (2.83)  32.96 (17.95)  32.36 (20.90)  22.53 (8.37) | 45  20  3  13  31  9 | 31.11 (11.03)  31.65 (15.10)  23.00 (8.19)  35.38 (10.81)  27.71 (11.51)  37.78 (13.75) | 22.79 (16.39)  20.62 (15.28)  13.61 (5.81)  27.76 (15.25)  18.62 (15.56)  27.48 (21.13) |
| By child’s sex  Males  Females | 27  40 | 32.00 (13.74)  35.35 (22.58) | 29.46 (15.49)  33.22 (22.79) | 6  61 | 35.17 (17.66)  30.92 (11.64) | 30.13 (28.14)  21.23 (14.19) |
| By Form Version  Preschool  School Age | 0  67 | 34.00 (19.45) | 31.71 (20.12) | 1  66 | 49.00  31.03 (12.07) | 41.20  21.73 (15.74) |

^1^ Total SRS score as predicted by the CAT (based on the subset of items administered in the CAT only). ^2^Scaled per equipercentile equating method.

**Figure S3.** Within-subjects comparison of full 65-item short SRS scores.

**a)**


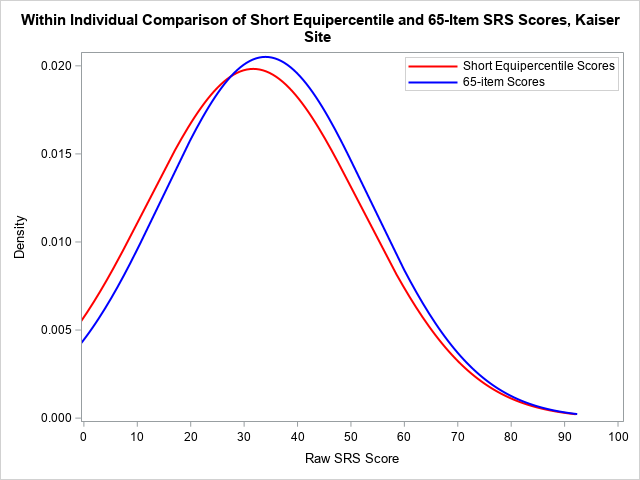


***b)***


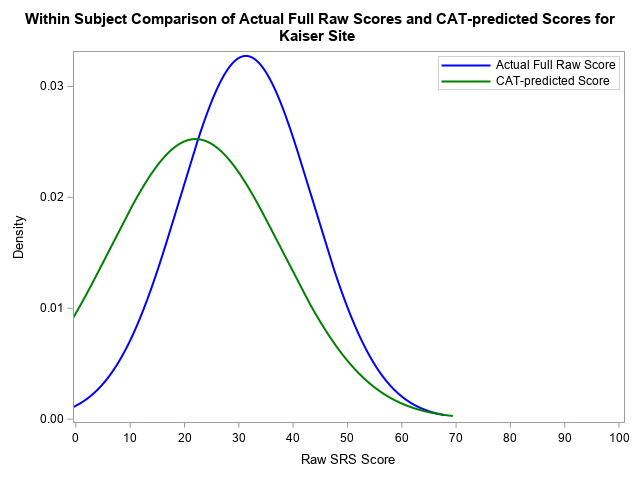


Figure legend: Normal density plot of within-individual score comparisons from participants from the general population site for the (a) short version group; short scores scaled to raw total full scores using equipercentile equating; and for the (b) CAT version group; CAT scores scaled using predicted CAT scores as described in text.

**Figure S4.** Within-subjects comparison of full and short and full and CAT-predicted SRS scores.

**a)**

**
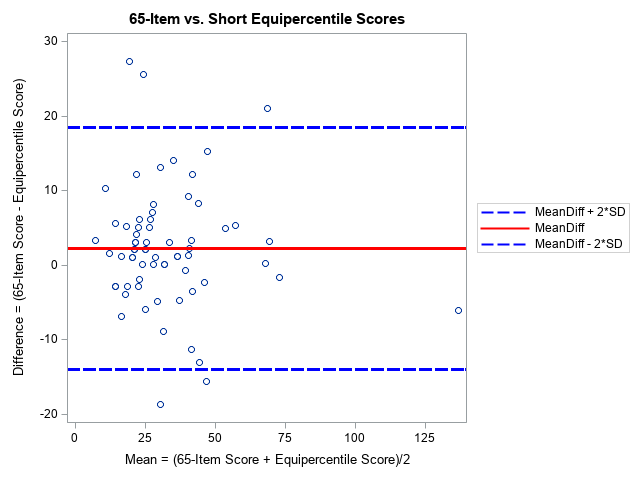
**

**b)**


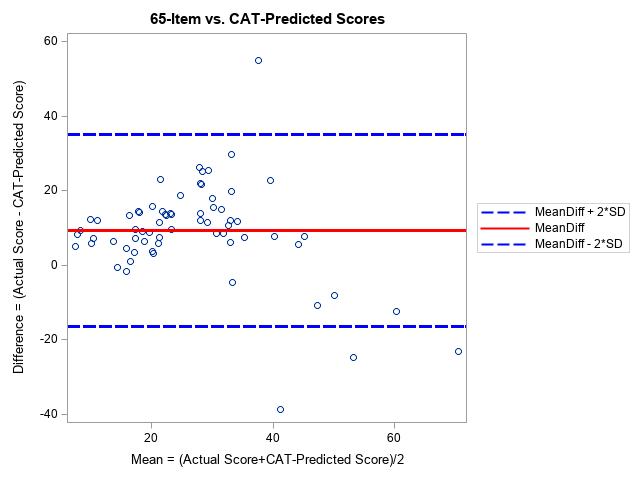


Figure legend: Bland-Altman plots comparing mean difference in scores between full and shortened version SRS scores for participants from the general population site, who were administered “remaining” SRS questions not part of shortened versions due to their participation in another study. a) Comparison of full 65-item SRS scores (raw total) and short 16 item scores (equipercentile scaled) for participants in the short SRS group; and (b) Comparison of full 65-item scores (raw total) SRS scores and CAT- SRS scores (CAT-predicted raw total) for participants in the CAT group.

**Figure S5**. ROC curve predicting ASD status by SRS administration method and SRS form.

***a)***


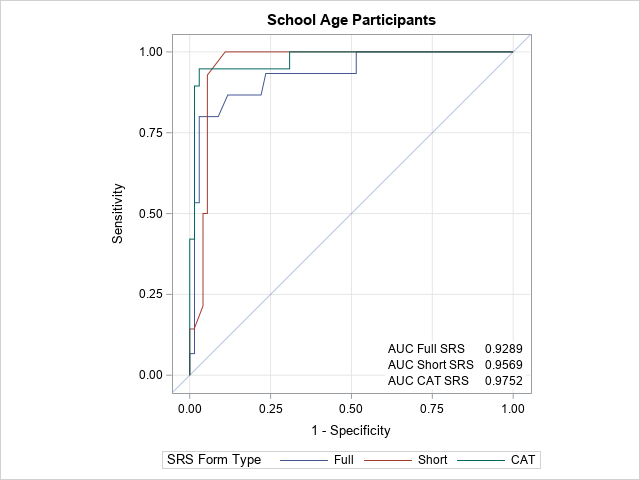


SRS Version

***b)***


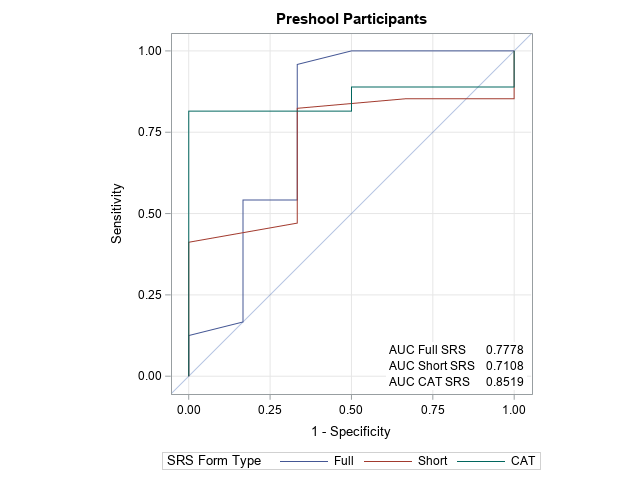


SRS Version

*Figure legend:* ROC Curves are shown for full 65-item raw total SRS scores (in blue), 16-item short SRS scores (scaled via equipercentile equating, shown in red), and CAT-SRS scores (CAT-predicted, shown in green) from analyses predicting ASD diagnosis including all ASD cases. AUC values are shown in the lower right-hand corner. Data was combined across sites, given low number of cases from the general population site, and relatively few non-cases from the clinical site. One individual from the CAT group is not included due to a missing CAT-predicted score and another is not included due to inconclusive diagnostic status. Plots are shown in (a) participants administered school aged forms, n=257, and (b) participants administered preschool forms, n=96

**Table S5. Area under the curve (AUC) values and cutoff points for SRS full, short, and CAT score ROC analyses in association with ASD diagnosis**

|  | **Full SRS** | | | | | | **Short SRS** | | | | | | **CAT SRS** | | | | | |
| --- | --- | --- | --- | --- | --- | --- | --- | --- | --- | --- | --- | --- | --- | --- | --- | --- | --- | --- |
|  | N | AUC | Raw Score cutoff^1^ | T-score^2^ | Sens | Spec | N | AUC | Scaled Score cutoff^3^ | T-score | Sens | Spec | N | AUC | CAT-predicted Score cutoff | T-score | Sens | Spec |
| **Total ROC analytic sample** | 113 | 0.9245 | 36.00 | 51, 53 | 0.97 | 0.74 | 124 | 0.9057 | 70.70 | 64,67 | 0.81 | 0.93 | 116 | 0.9655 | 68.68 | 64, 66 | 0.89 | 0.96 |
| **Child Sex** |  |  |  |  |  |  |  |  |  |  |  |  |  |  |  |  |  |  |
| Male | 70 | 0.8784 | 60.00 | 60 | 0.71 | 0.90 | 66 | 0.9324 | 61.40 | 61 | 0.91 | 0.91 | 43 | 0.8786 | 87.53 | 72 | 0.83 | 0.88 |
| Female | 43 | 0.9915 | 51.00 | 59 | 0.91 | 1.00 | 58 | 0.8442 | 73.60 | 69 | 0.71 | 0.93 | 73 | 0.9721 | 76.51 | 70 | 0.91 | 1.00 |
| **SRS form** |  |  |  |  |  |  |  |  |  |  |  |  |  |  |  |  |  |  |
| School-age | 83 | 0.9289 | 60.00 | 60, 63 | 0.80 | 0.97 | 87 | 0.9569 | 67.80 | 64, 66 | 1.00 | 0.89 | 87 | 0.9752 | 76.51 | 67, 70 | 0.95 | 0.97 |
| Preschool | 30 | 0.7778 | 38.00 | 49 | 0.96 | 0.67 | 37 | 0.7108 | 58.00 | 56 | 0.82 | 0.67 | 29 | 0.8519 | 76.53 | 64 | 0.81 | 1.00 |
| **Non-ASD outcomes** | 74 | 0.5868 | 32.00 | 49, 51 | 0.56 | 0.66 | 76 | 0.7536 | 31.90 | 49, 51 | 0.87 | 0.61 | 70 | 0.6653 | 17.45 | 43, 45 | 0.77 | 0.58 |

Abbreviations as follows: AUC= Area Under the Curve; Sens=sensitivity; Spec=Specificity; ROC=Receiver Operating Characteristic. ^1^Raw scores rounded up to closest whole number for T score norm comparisons. Cut off scores shown provide the maximum sensitivity; for several of the scores in the 30s, secondary cut-offs of 50 yielded lower sensitivity and somewhat higher specificity (eg, total sample, full SRS, secondary raw cut off score of 50 yielded a sensitivity of 0.85 and specificity of 0.86). ^2^T scores shown are male, female where not stratified by sex and from school-age form norms for all rows but the preschool form row. Preschool T-score norms are not provided by sex. ^3^Scaled according to equipercentile equating as described in the text.

**Table S6**. Descriptive statistics of POMP-scaled SRS scores^1^ by administration method and study site

|  | **Clinical Site** | | | | **General Population Site** | | | |
| --- | --- | --- | --- | --- | --- | --- | --- | --- |
|  |  | Full  (n=46) | Short^1^  (n=58) | CAT^2^  (n=50) |  | Full  (n=67) | Short^1^  (n=67) | CAT^2^  (n=67) |
|  | n | Median (IQR) | | | n | Median (IQR) | | |
| Overall | 154 | 34.10 (27.69) | 47.92 (27.08) | 46.23 (25.64) | 201 | 13.85 (9.23) | 8.33 (10.42) | 20.00 (15.58) |
| ASD Diagnosis^2^  Yes  No | 130  23 | 36.41 (25.13)  29.23 (21.54) | 48.96 (22.92)  33.33 (50.00) | 46.30 (25.86)  27.54 (32.58) | 4  197 | 60.00 (3.08)  12.82 (8.72) | 58.33 (41.67)  8.33 (10.42) | -  20.00 (15.58) |
| Race/ethnicity  Non-Hispanic  Hispanic  Unknown Ethnicity  Black or African American  Asian  White  Hawaiian or Pacific Islander  Biracial or Multiracial  Other Race  Unknown Race | 106  31  17  80  11  34  1  6  5  17 | 33.33 (29.23)  52.05 (15.38)  32.82 (23.59)  33.33 (30.77)  30.51 (13.33)  33.59 (32.82)  -  39.74 (6.67)  -  56.41 (6.15) | 41.67 (33.33)  54.17 (37.50)  50.00 (30.21)  42.71 (27.08)  48.96 (27.08)  54.17 (31.25)  -  45.83 (54.17)  41.67  52.08 (28.13) | 44.87 (24.90)  44.17 (34.87)  46.48 (18.37)  46.15 (25.56)  63.33  43.33 (24.71)  22.22  29.17 (8.33)  67.61 (32.30)  46.48 (2.69) | 138  59  4  7  22  129  2  17  2  22 | 12.31 (9.23)  13.85 (8.21)  -  7.95 (0.51)  17.95 (15.38)  12.31 (8.72)  24.10  13.85 (2.56)  -  19.49 (15.90) | 8.33 (10.42)  15.63 (8.33)  3.13 (6.25)  7.29 (2.08)  8.33 (10.42)  10.42 (10.42)  2.08  6.25 (8.33)  16.67 (16.67)  11.46 (23.96) | 21.57 (14.81)  18.18 (7.73)  25.00 (27.78)  15.94 (16.67)  28.21 (19.09)  19.44 (15.51)  -  18.18 (9.85)  -  20.00 (19.32) |
| By child’s sex  Males  Females | 112  42 | 34.36 (30.77)  32.82 (24.62) | 43.75 (25.00)  50.00 (54.17) | 46.67 (26.08)  40.19 (20.04) | 68  133 | 14.87 (11.79)  12.82 (5.38) | 8.33 (10.42)  10.42 (10.42) | 25.09 (11.76)  19.70 (12.42) |
| By Form  Preschool  School age | 97  57 | 35.64 (29.23)  34.10 (25.90) | 45.83 (31.25)  52.08 (36.46) | 43.33 (25.31)  47.22 (26.38) | 1  200 | -  13.85 (9.23) | -  8.33 (10.42) | 53.33  20.00 (12.42) |

^1^SRS scores for all administration methods scaled from 0 to 100 using the % of maximum possible (POMP) method.

^2^Diagnosis for one participant was inconclusive and not included in ASD comparisons.

**Figure S6.** POMP-scaled SRS Score Distributions by Administration Method.

**a)** Drexel Site


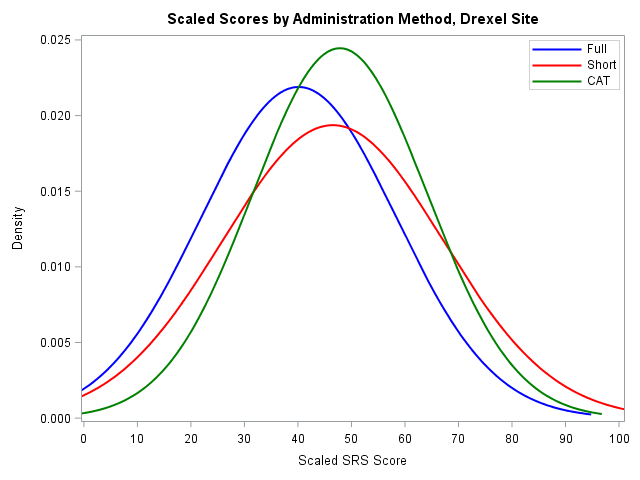


**b)** Kaiser Site


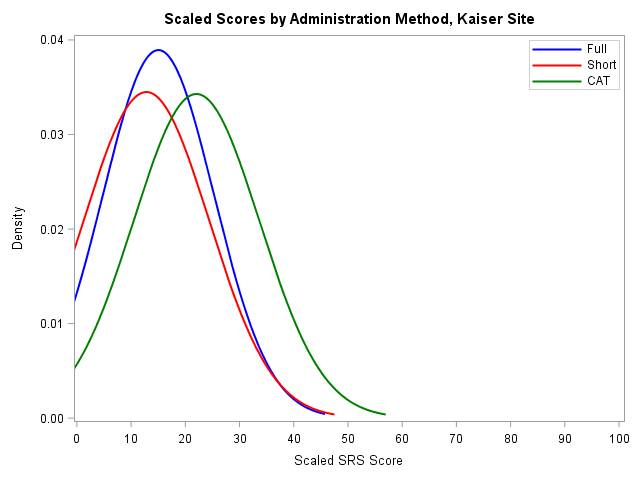


**Figure S7.** ROC curve predicting other non-ASD neurodevelopmental or psychiatric outcomes by SRS administration method.

SRS Version


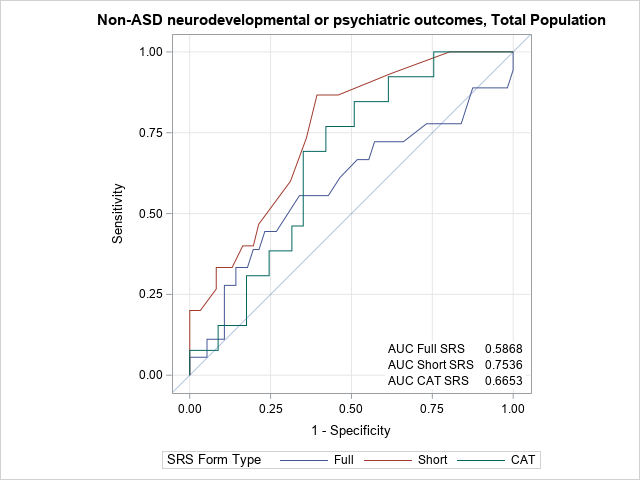


*Figure legend:* ROC Curves are shown for full 65-item raw total SRS scores (in blue), 16-item short SRS scores (scaled via equipercentile equating, shown in red), and CAT-SRS scores (CAT-predicted, shown in green) from analyses predicting non-ASD neurodevelopmental or psychiatric outcomes indicated in clinical or medical records, which included speech delay, seizure disorder, anxiety, ADHD, cerebral palsy, conduct disorder, adjustment disorder, and motor delay, or other developmental delay. A total of 220 participants were included in this analysis (135 individuals with ASD or for whom ASD status was unknown, including 5 with both ASD and other diagnoses, were not included here). Of those with a non-ASD diagnosis, 18 participants received the full SRS, 15 received the short SRS, and 13 received the CAT SRS.

Table S7. Subscale item content and structure in full vs shortened measures

| Subdomain | Original  65 item | Sturm et al  16 item  “Short form” | Generation R 18 item (for comparison)^1^ |
| --- | --- | --- | --- |
| Social Awareness | 12% | 13% | 0% |
| Social Communication | 18% | 44% | 44% |
| Social Cognition | 34% | 13% | 28% |
| Social Motivation | 17% | 6% | 0% |
| Autistic Mannerisms | 18% | 25% | 28% |
| *Reverse coded items* | 26% | 19% | 11% |

^1^The Generation R study uses an 18-item investigator derived subset of the SRS (as a stand-alone questionnaire).

Table S8. Basic characteristics of study participants by site and COVID time period (n=355)

|  | Clinical Site | | General Population Site | |
| --- | --- | --- | --- | --- |
| Characteristic^1^ | Pre-COVID  n=127 | COVID  n=27 | Pre-COVID  n=146 | COVID  n=55 |
| Child’s Age (yrs) mean (SD) | 3.5 (0.7) | 3.54 (0.8) | 4.0 (0.2) | 4.1 (0.3) |
| Child’s Sex |  |  |  |  |
| Male  Female | 93 (73%)  34 (27%) | 19 (70%)  8 (30%) | 67 (46%)  79 (54%) | 1 (2%)  54 (98%) |
| Informant’s relationship to child |  |  |  |  |
| Mother | 104 (82%) | 23 (85%) | 146 (100%) | 55 (100%) |
| Other guardian/caregiver | 21 (16%) | 3 (11%) |  |  |
| Unknown | 2 (2%) | 1 (4%) |  |  |
| Mother’s Race |  |  |  |  |
| White or Caucasian | 26 (20%) | 8 (30%) | 106 (73%) | 23 (42%) |
| Black or African-American | 67 (53%) | 13 (48%) | 4 (3%) | 3 (5%) |
| Asian | 11 (9%) |  | 10 (7%) | 12 (22%) |
| Biracial or Multiracial | 5 (4%) | 1 (4%) | 8 (5%) | 9 (16%) |
| Hawaiian or Pacific Islander |  | 1 (4%) | 2 (1%) |  |
| Other | 1 (1%) | 4 (15%) | 2 (1%) |  |
| Unknown | 17 (13%) |  | 14 (10%) | 8 (15%) |
| Mother’s Ethnicity |  |  |  |  |
| Hispanic | 24 (19%) | 7 (26%) | 44 (30%) | 15 (27%) |
| Non-Hispanic | 86 (68%) | 20 (74%) | 98 (67%) | 40 (73%) |
| Unknown | 17 (13%) |  | 4 (3%) |  |
| Mother’s Education |  |  |  |  |
| HS degree or less | 40 (31%) | 9 (33%) | 6 (4%) | 1 (2%) |
| Some College/trade school | 37 (29%) | 7 (26%) | 43 (30%) | 14 (29%) |
| College Degree | 23 (18%) | 10 (37%) | 59 (40%) | 19 (35%) |
| Post-College Degree | 13 (10%) | 1 (4%) | 32 (22%) | 18 (33%) |
| Unknown | 14 (11%) |  | 6 (4%) | 1 (2%) |
| ASD Diagnosis |  |  |  |  |
| Yes | 106 (83%) | 24 (89%) | 4 (3%) |  |
| No | 21 (17%)^2^ | 3 (11%) | 142 (97%) | 55 (100%) |
| SRS Form |  |  |  |  |
| Preschool | 84 (66%) | 13 (48%) |  | 1 (2%) |
| School-age | 43 (34%) | 14 (52%) | 146 (100%) | 54 (98%) |
| SRS version/administration method  Full 65-item SRS  Short 16-item SRS  CAT SRS | 46 (36%)  58 (46%)  23 (18%) | 27 (100%) | 67 (46%)  67 (46%)  12 (8%) | 55 (100%) |

Abbreviations: ASD, Autism Spectrum Disorder; SRS, Social Responsiveness Scale.

^1^N(%) shown unless indicated.

^2^1 individual had inconclusive diagnostic status following evaluation and was included in the ‘no’ category.

Table S9. Mean (SD) scores by site and COVID time period.

|  | Drexel - CAT | | Kaiser-CAT | |
| --- | --- | --- | --- | --- |
|  | n | Mean (SD) | n | Mean (SD) |
| Pre-COVID | 23 | 96.02 (32.8) | 12 | 19.66 (20.9) |
| COVID | 26^1^ | 105.83 (34.2) | 55 | 22.54 (14.6) |

Table displays only CAT scores given only CAT administrations were impacted by COVID, due to our block randomization scheme.

^1^One individual missing CAT-predicted score

**Appendices**

Appendix S1. SRS Short Form questions and subscales

| **Question** | **Treatment Subscale** | **Description** |
| --- | --- | --- |
| q4 | Autistic Mannerisms | Inflexible in stressful situations |
| q7 | Social Awareness | Challenges in perceiving what others think and feel |
| q8 | Autistic Mannerisms | Exhibits behaviors that could be perceived as strange or bizarre |
| q13 | Social Communication | Struggles to take turns with peers (e.g. in conversation) |
| q16 | Social Communication | Makes poor eye contact |
| q18 | Social Communication | Challenges in making friends |
| q22 | Social Communication | Difficulty engaging in play with peers |
| q23 | Social Motivation | Unmotivated to engage in group activities |
| q29 | Autistic Mannerisms | Perceived as weird by peers |
| q30 | Social Cognition | Emotionally distraught in busy situations |
| q33 | Social Communication | Exhibits social awkwardness |
| q37 | Social Communication | Challenges in relating to other children |
| q38 | Social Communication | Challenges in responding to others' mood changes |
| q39 | Autistic Mannerisms | Range of interests is narrow |
| q42 | Social Cognition | Displays hypersensitivity to auditory, olfactory or tactile stimuli |
| q54 | Social Awareness | Treats people like objects |

*Item content is summarized in description and not copied due to copyright. Color coding is used to differentiate subscales.*

**Appendix S2. Computer-adaptive testing (CAT) SRS development methodology.**

A brief description of CAT development and methodology is provided in the text. In addition, further details on CAT development are provided in Kaat et al (submitted manuscript).

**CAT-SRS development**

Item response theory analyses were conducted using two primary data sources: existing data from 11 cohorts from the Environmental Influences on Child Health Outcomes (ECHO) program, as previously described in Lyall et al, 2021 (PMCID: PMC7965796), as well as additional data from large sibling studies as described in Mulligan et al, 2015 (PMCID: PMC4489415). A model-building approach was used, first using school-aged form data and examining dimensionality of the items and testing for differential item functioning (DIF) by sex assigned at birth. DIF by age was also examined for identical items across preschool and school-age forms. Final calibrations of the SRS were obtained using a multiple-group IRT model. The graded response model (GRM) was used for item calibrations (Samejima, 2016). This model includes slope parameter (or more when extending to the multidimensional GRM) and one fewer threshold or intercept parameters than there are response options for an item. The slope parameter represents how strongly an item is related to the underlying construct and a threshold parameter represents the point along the latent construct at which the respondent has a 50 % chance of picking that category or any higher than it.

For evaluation of DIF, hybrid ordinal logistic regression was used (Choi et al., 2011; Crane et al., 2006; Crane et al., 2007), regressing item responses on a conditional score (the expected IRT score from a GRM model after purification of anchor items), group membership for the putative DIF variable, and the interaction between group and conditioning score. Items were flagged for potential DIF based on the statistical significance of nested chi-square tests between the hybrid ordinal logistic regression models, further purifying the potential anchor items in the conditioning score until two series of analyses flagged the same set of potential DIF items (Choi et al., 2011).

After this model building process had selected items from the SRS, a multiple-group IRT model with the mirt package in R was conducted (Chalmers, 2012). To set the scale for IRT-based score, the distribution was fixed (mean=0, sd=1) among males taking the school-age form. Then items that were common across forms that did not exhibit DIF were equated across groups, and all items within a form were equated across groups, such that items could be categorized as invariant within the preschool form, invariant within the school-age form, and invariant across both forms. In this way, the latent distribution for all other groups could be estimated on the same scale as the centering sample.

CAT-SRS implementation

The item with the maximum information content as determined by IRT analyses described above was selected as the starting item (Item 35). As summarized in the text, administration stopped when either all 65 items had been administered, or the minimum number of items was administered (n=10) and at least one of the following conditions was also met: a) the empirical reliability was > 0.90 (SE < 0.3) or 2b) when the score reached equilibrium, as indexed by the change in EAP score estimates [with the diffuse prior] between two consecutive items not exceeding 0.05 (i.e., |ΔEAP| < 0.05, which is in standard deviation units).

R code to administer the CAT is available on GitHub or by contacting the authors; additional files required to run the program include an item content file and a parameter file, which cannot be copied here due to publisher provisions from sharing item content.

Appendix S3: Equipercentile equated scores for scaling short 16-item SRS scores to full 65-item SRS scores

| SRS2-16 | SRS2 | Bootstrapped SE |
| --- | --- | --- |
| 0 | 5.7 | 0.17 |
| 1 | 11.4 | 0.23 |
| 2 | 15.8 | 0.24 |
| 3 | 19.9 | 0.26 |
| 4 | 23.9 | 0.29 |
| 5 | 27.9 | 0.34 |
| 6 | 31.9 | 0.39 |
| 7 | 35.8 | 0.44 |
| 8 | 39.7 | 0.51 |
| 9 | 43.5 | 0.59 |
| 10 | 47.3 | 0.68 |
| 11 | 51 | 0.79 |
| 12 | 54.6 | 0.93 |
| 13 | 58 | 1.09 |
| 14 | 61.4 | 1.28 |
| 15 | 64.6 | 1.49 |
| 16 | 67.8 | 1.7 |
| 17 | 70.7 | 1.91 |
| 18 | 73.6 | 2.1 |
| 19 | 76.4 | 2.28 |
| 20 | 79.1 | 2.43 |
| 21 | 81.8 | 2.56 |
| 22 | 84.4 | 2.68 |
| 23 | 87.1 | 2.79 |
| 24 | 89.8 | 2.92 |
| 25 | 92.6 | 3.07 |
| 26 | 95.5 | 3.26 |
| 27 | 98.5 | 3.48 |
| 28 | 101.7 | 3.75 |
| 29 | 105.1 | 4.05 |
| 30 | 108.6 | 4.38 |
| 31 | 112.2 | 4.74 |
| 32 | 116 | 5.12 |
| 33 | 119.8 | 5.51 |
| 34 | 123.8 | 5.91 |
| 35 | 127.8 | 6.31 |
| 36 | 131.8 | 6.7 |
| 37 | 135.9 | 7.09 |
| 38 | 140 | 7.48 |
| 39 | 144.1 | 7.86 |
| 40 | 148.1 | 8.22 |
| 41 | 152.2 | 8.58 |
| 42 | 156.3 | 8.92 |
| 43 | 160.3 | 9.24 |
| 44 | 164.4 | 9.55 |
| 45 | 168.4 | 9.95 |
| 46 | 172.5 | 10.5 |
| 47 | 176.6 | 10.87 |
| 48 | 181.5 | 10.96 |

16 item scores range from a possible total score of 0-48. Scores were equated by determining the equivalent percentile of the short score to the corresponding full score.
